# An Online Method to Detect and Locate an External Load on the Human Body with Applications in Ergonomics Assessment

**DOI:** 10.3390/s20164471

**Published:** 2020-08-10

**Authors:** Marta Lorenzini, Wansoo Kim, Elena De Momi, Arash Ajoudani

**Affiliations:** 1 ^1^HRI^2^ Laboratory, Istituto Italiano di Tecnologia, 16163 Genoa, Italy; marta.lorenzini@iit.it (M.L.); arash.ajoudani@iit.it (A.A.); 2 Department of Electronics, Information and Bioengineering, Politecnico di Milano, 20133 Milan, Italy; elena.demomi@polimi.it

**Keywords:** novel methods and systems for integrated ergonomic assessment, monitoring human-environment interaction, health monitoring in working environments

## Abstract

In this work, we propose an online method to detect and approximately locate an external load induced on the body of a person interacting with the environment. The method is based on a torque equilibrium condition on the human sagittal plane, which takes into account a reduced-complexity model of the whole-body centre of pressure (CoP) along with the measured one, and the vertical component of the ground reaction forces (vGRFs). The latter is combined with a statistical analysis approach to improve the localisation accuracy, (which is subject to uncertainties) to the extent of the industrial applications we target. The proposed technique eliminates the assumption of known contact position of an external load on the human limbs, allowing a more flexible online body-state tracking. The accuracy of the proposed method is first evaluated via a simulation study in which various contact points on different body postures are considered. Next, experiments on human subjects with three different contact locations applied to the human body are presented, revealing the validity of the proposed methodology. Lastly, its benefit in the estimation of human dynamic states is demonstrated. These results add another layer to the online human ergonomics assessment framework developed in our laboratory, extending it to more realistic and varying interaction conditions.

## 1. Introduction

In recent years, an ever-growing number of small- and medium-sized companies are adapting their industrial processes to the demands of the contemporary market with high-mix and low-volume production [1]. In such processes, human workers have to operate in, and interact with environments which vary continuously and dynamically. These new working conditions make the traditional view of occupational ergonomics in the design of the static workplaces less valid and hardly applicable [2].

The tools to assess human factors in the brand-new industrial background should provide quantitative evaluation and anticipation of the human psycho-physical conditions and delivered effort throughout the working activity. This entails the development of modular sensory systems and human kinodynamic states estimation algorithms, which can adapt to the varying tasks involved in the working process and to the ever-changing interactions of humans with the environment. Previous works to address this focused on the development of human dynamic models [3,4,5,6], to detect internal body states (e.g., joint torques) under known body postures and external forces. However, from a practical standpoint, one of the open challenges is the ability to identify the application point of an external force such as the weight of a load on the workers’ body using wearable or suitable-for-industry sensory systems.

Several approaches can be found in literature for detecting the contact point location of an external force acting on a body. Drawing inspiration from the robotics community, in [7], the total energy and the generalized momentum of the robot manipulator are used to develop a collision detection method that uses only proprioceptive robot sensors and provides even directional information to make the robot safely react after the collision. On the basis of the same principles, in [8] a physical collision detection/reaction technique is presented, based on a residual signal which needs only joint position measures, which can be given by the encoders. However, certain quantities cannot just be obtained for humans thus none of these solutions are practically feasible. On the other hand, some researches [9,10] show that a possible approach consists in employing additional sensors acting as a skin on the robot body. Nevertheless, this solution is not feasible for humans since it may limit mobility and cause frustration. In fact, when dealing with humans, alternative approaches involving more practical and less invasive systems need to be used.

A physics-based optimization procedure, merged with a machine learning technique is proposed in [11] to estimate forces exerted by human participants in multi-contact interaction with rigid environments. The method uses motion-capture only, but it can just be implemented in an off-line phase. Other approaches explore the use of computer vision technology [12,13] to infer interaction forces. In this direction, the contact points are observed from the image data alone and but this is often too ambiguous and noisy to reconstruct subtle interactions and contact phenomena between the hand and an object. In [14], a technique to directly estimate the external forces in real-time by combining inverse dynamics computation and a physiological muscle model is developed. Nevertheless, this approach requires the use of electromyography (EMG) sensors, which besides being unpractical in the industrial settings we target, present several drawbacks [15] considering the correct placement of the sensors, noise signals or artifacts, movement of the electrodes in dynamic conditions, etc.

Accordingly, the main objective of this work is to fill in the gap between the current insufficient human ergonomics monitoring systems in industry, which are pen-and-paper-based observational methods (e.g., Ergonomic Assessment Work-Sheet (EAWS)), and the complex, hardly personalisable laboratory models, which exploit less reliable bio-signal measurements such as EMGs. In this direction, we propose a novel method for contact point detection and localisation which can be successfully applied to humans using solely practical and light-weight sensory systems. The method is based on a torque equilibrium condition on the human sagittal plane, which takes into account a reduced-complexity model of the whole-body centre of pressure (CoP), identified in an off-line phase, along with the measured CoP, and the vertical component of the ground reaction forces (vGRFs). The latter is combined with a statistical analysis approach to improve the localisation accuracy (which is subject to sensor noise and uncertainties in the model) to the extent of the industrial applications we target.

The second objective of this work is to extend the online estimation algorithm we proposed in [16] to account for the overloading torques induced into the human body joints by an external load. This eliminates the assumption of a fixed and predefined contact point (i.e., the human hand/s holding an object) that was made so far within the procedure. Hence, this extension increases the potential of the ergonomics assessment method (e.g., monitoring of the physical loading induced on human joints by an external object/tool) presented in [16] while improving its flexibility to varying interaction conditions in realistic industrial settings.

**Figure 1:**
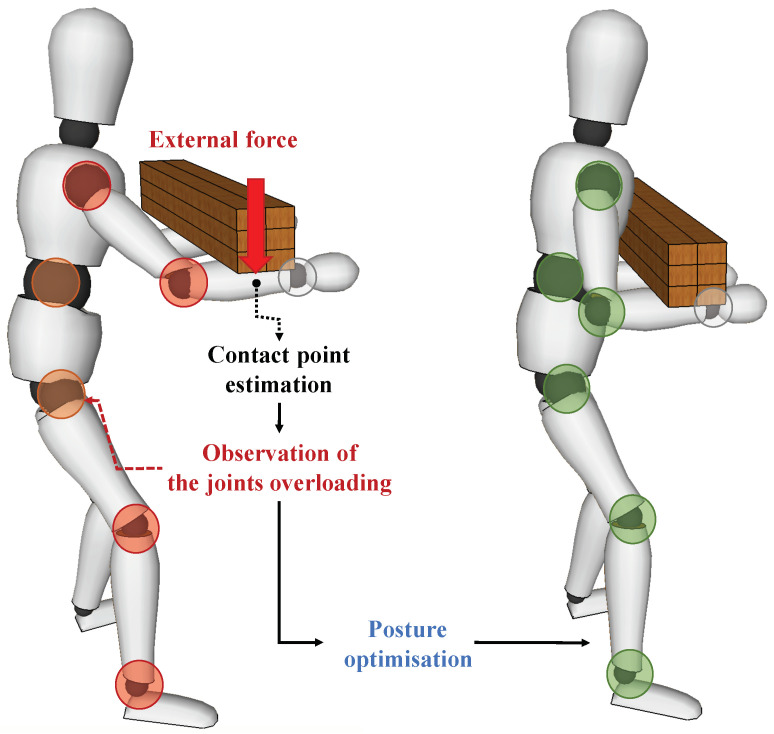
In this work we propose a contact point detection and localisation method which can be applied in the estimation of the torques induced on human joints by an external load. By minimising such torques, the human body posture can be optimised, leading to a more ergonomic behaviour.

Subsequent to these advancements, an optimisation of the human posture by minimising such overloading torques is presented. As a result, human workers can achieve more ergonomic and comfortable body configurations where the risk of human joint injuries can be significantly reduced. The whole framework is represented in Figure 1.

The capability of the contact point detection technique to localise the application point of an external load is tested through a simulation study using Simscape Multibody™. Then, an experimental analysis on two human subjects is made to understand its potential in realistic settings. First, a validation of the proposed method is performed considering three different locations of a load on the body. Then, its application to the estimation of human dynamic states is presented. In this work, “human dynamic states” refer to those variables, measurable on the humans, that are determined by the intervention of a force as the loading induced on the human joints by an external force. The contact point position is first detected and the overloading torques induced by the external load are computed accordingly. The latter can then be used for the assessment and optimisation of the human body posture.

## 2. Contact Point Detection and Localisation

In this section, we introduce a novel approach to detect and locate the contact point of an external load applied to the human body. First, we provide an analytical method to compute it by means of the measured and the estimated human CoP and GRFs. Next, we present the human model employed in this work to obtain the CoP estimation, which is based on the statically equivalent serial chain (SESC) technique [17].

### 2.1. Estimation of the Contact Point Position

A floating base model of the human body is developed in this work. The human pelvis frame is set as the base frame $\Sigma_{0}$ attached to the inertial frame $\Sigma_{W}$ through six virtual degrees of freedom (DoFs). The generalised coordinates of the system are defined by $q={\left[\begin{array}{ccc} x_{0}^{T} & \theta_{0}^{T} & q_{h}^{T} \end{array}\right]}^{T}\in R^{6+n}$. $x_{0}^{T}$ and $\theta_{0}^{T}$ represent the position and the orientation of $\Sigma_{0}$, respectively. $q_{h}\in R^{n}$ denotes the angular position of human joints and *n* is the number of joints. Assuming $n_{c}$ contact forces vector $f_{i}\in R^{6}$ applied on the segments, the equation of motion in the joints space can be written as

(1) $$S^{T}\tau =\tau_{b}-\sum_{i=1}^{n_{c}}J_{p_{i}}^{T}f_{i},$$

where $\tau_{b}=M\ddot{q}+C\dot{q}+G\in R^{n+6}$ and M, C and G represent the inertia matrix, the vector of centrifugal and Coriolis forces, and the vector of the gravity force, respectively. $S=\left[\begin{array}{cc} 0_{n\times 6} & I_{n\times n} \end{array}\right]\in R^{n\times (n+6)}$ is the actuation matrix, $\tau \in R^{n}$ is the vector of applied joint torques, and $J_{p_{i}}\in R^{6\times (n+6)}$ is the contact Jacobian at the point $p_{i}$ with respect to $\Sigma_{W}$. By employing Equation (1), given $J_{p_{i}}$ and $f_{i}$, it is possible to address the overloading effect induced on the joints of a human which is performing a manipulation task interacting with a tool or an object [16].

**Figure 2:**
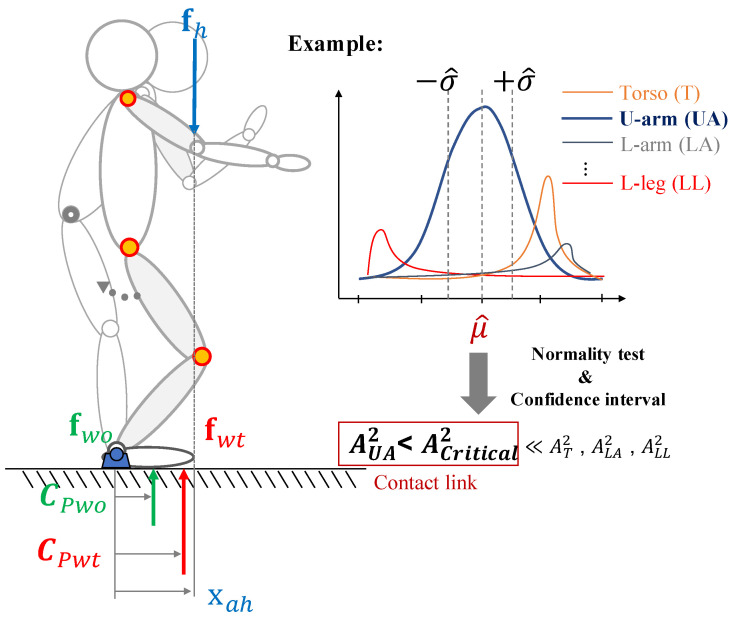
The overall procedure to detect the contact point starting from a torque equilibrium condition.

To obtain the contact point position in the human body link, a torque equilibrium condition can be considered, which is approximated by an equivalent mechanical system performing quasi-static movements (naturally arising when executing heavy manipulation tasks). In general, an interaction force vector F that acts on a rigid body system results in the joint torques vector T as

(2) $$T=x_{p}\times F,$$

where $x_{p}$ represents the contact point vector on the body. Due to the multiple interaction forces, several torques are acting on the body at the same time but the body stays at rest. It means that there is no net torque ($T_{net}=0$) acting on the object, which is hence in an equilibrium condition. Similarly, let us consider the interaction forces acting on the body on the left side of Figure 2.

The torques are generated by the weight of an external object, thus, the equilibrium condition is

(3) $$C_{P_{wt}}\times f_{wt}=C_{P_{wo}}\times f_{wo}-x_{a_{h}}\times f_{h},$$

where $C_{P_{wt}}$ and $C_{P_{wo}}$ are the body CoP in the loaded and not loaded condition, respectively. $f_{wt}$ is the vGRF vector applied at $C_{P_{wt}}$, which is obtained from the combined mass of the human body and the external object/tool, while $f_{wo}$ depends only on the human body. $f_{h}$ represents the weight of the object/tool that is applied at the contact point $x_{a_{h}}$. By introducing the assumption of single contact point, the weight of the object/tool can be defined as $f_{h}=-\left(f_{wt}-f_{wo}\right)$. Since the vGRF on a flat surface (typical condition within industrial work spaces) is much larger than the tangential components of the ground reaction force, the latter can be neglected and the vGRF can be represented by the human body weight, which is acting on the projected CoP position [18].

Hence, considering the forces and points projected onto the ground, for the human in the sagittal plane, the moment equilibrium equation about a pivot (i.e., ankle joint) can be written as

(4) $$x_{a_{h}|x}=\frac{C_{P_{wt}|x}f_{wt}-C_{P_{wo}|x}f_{wo}}{\left(f_{wt}-f_{wo}\right)},$$

where $x_{a_{h}|x}$ is the *x*-coordinate of the load application point (i.e., contact point). The whole-body CoP vector $C_{P_{wo}}$ is achieved by a human-body model, which will be explained in Section 2.2. On the other hand, the CoP vector $C_{P_{wt}}$ can be measured by a sensor system.

The link, which includes the contact point, must now be identified. The procedure employed in this work is explained below and the corresponding pseudocode is presented in Algorithm 1.

**Algorithm 1** Detection of contact point position.

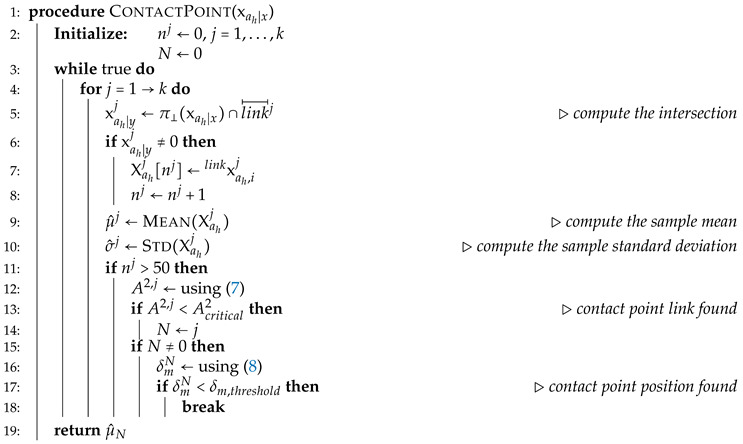



First, the algorithm starts to determine whether the point of intersection between a *j*-th link segment $\overset{⟝\;⟞}{{link}^{j}}$ (with link index $j\in [\begin{array}{ccc} 1 & \cdots & k \end{array}]$) and $\pi_{⊥}\left(x_{a_{h}|x}\right)$, which is the perpendicular line passing through $x_{a_{h}|x}$, exists. In fact, it is possible to obtain multiple values for the load application point on *y*-axis ($x_{a_{h}|y}$) when the perpendicular line $\pi_{⊥}\left(x_{a_{h}|x}\right)$ intersects more than one link along its path. Every time the intersection point ${}^{link}x_{a_{h}}^{j}$ w.r.t. $\Sigma_{link}$ is computed for the *j*-th link, its measurement is collected and stored in the vector $X_{a_{h}}^{j}$.

Next, to calculate the contact point which comes closer to the most likely value, by considering the mean value of the data set in $X_{a_{h}}^{j}$, we use a statistical hypothesis test for normality. It should be noted that the estimation of the load application point, made by using (4), is based on the measurements collected with a motion-capture system and a force sensor. Hence, some disturbances due to sensors noise are inevitably introduced during the estimation process. Furthermore, when dealing with humans, the artifacts generated by their natural ever-present movement must be taken into account. To model the resulting error, the Gaussian model is widely used in inertial body tracking [19,20], but also in the characterisation of the human movement [21]. Similarly, the data set that we build can be described as [22,23]

(5) $$X_{a_{h}}^{j}∼N\left({\hat{\mu }}^{j},{\hat{\sigma }}^{2,j}\right),$$

where ${\hat{\mu }}^{j}$ and ${\hat{\sigma }}^{j}$ are the mean value and the standard deviation of $X_{a_{h}}^{j}$, respectively, and N denotes the normal (i.e., Gaussian) distribution. Thus, we can assume that the approach to find a contact point from an approximate probability distribution is relying on the normality assumption. The one-sided Anderson-Darling test [24] is implemented online for this purpose. Prior to the test, the element $x_{a_{h},i}^{j}$ of $X_{a_{h}}^{j}$ for $i=1,\ldots ,n^{j}$ must be sorted such that $x_{a_{h},1}^{j}\leq x_{a_{h},2}^{j}\leq \ldots \leq x_{a_{h},n^{j}}^{j}$ and standardised as

(6) $$s_{i}^{j}=\frac{x_{a_{h},i}^{j}-{\hat{\mu }}^{j}}{{\hat{\sigma }}^{j}}.$$

First, a sufficient number of samples must be collected such that the condition $n^{j}>50$ is satisfied. Then, test statistic $A^{2,j}$ for the *j*-th link can be defined as

(7) $$A^{2,j}=-n^{j}-\sum_{i=1}^{n^{j}}\frac{2i-1}{n^{j}}\left[\ln\left(F\left(s_{i}^{j}\right)\right)+\ln\left(1-F\left(s_{n^{j}+1-i}^{j}\right)\right)\right].$$

where *F* is the standard normal cumulative distribution function and ln is the natural logarithm. If $A^{2,j}$ exceeds a given critical value, the hypothesis of normality is rejected with some significance level. The critical values, which depends on the number of samples and the significance level, can be found in [25]. Hence, the first link whose sample of contact points presents a value of $A^{2,j}$ under the critical value $A_{critical}^{2}$, is selected as the contact point link *N* (see Figure 2). This is because we expect the data set underlying the estimated contact point to be normally distributed.

Once the contact point link is detected, the contact point position ${}^{link}x_{a_{h}}^{N}$ w.r.t. $\Sigma_{link}$ must be calculated. At this point, to decide, whether or not, enough accuracy is reached by the collected sample, a method to monitor the estimated range of the desired parameter (i.e., contact point) is required. Due to the normality assumption previously made, the confidence interval of the sample mean can be employed to this aim. A confidence interval gives an estimated range of values which is likely to include an unknown population parameter, the estimated range being calculated from a given sample [26]. Since we consider the mean of the sample of the contact point link ${\hat{\mu }}^{N}$ to be equal to the estimated contact point position ${}^{link}x_{a_{h}}^{N}$, the magnitude of its confidence interval provides an estimate of the accuracy of the contact point computation. As soon as this confidence interval falls below a predefined threshold $\delta_{m,threshold}$, the sample mean ${\hat{\mu }}^{N}$ is selected as the contact point position. The confidence interval of the mean $\delta_{m}^{N}$ is computed as

(8) $$\delta_{m}^{N}=\pm z^{*}\frac{{\hat{\sigma }}^{N}}{\sqrt{n^{N}}}\;\;\;\mathrm{such}\;\mathrm{as}\;\;-z^{*}\frac{{\hat{\sigma }}^{N}}{\sqrt{n^{N}}}\leq {\hat{\mu }}^{N}\leq +z^{*}\frac{{\hat{\sigma }}^{N}}{\sqrt{n^{N}}},$$

where $z^{*}$ is a critical value which expresses the confidence level of the interval, which depends on the significance level, and can be found in [27].

### 2.2. Whole-Body Centre of Pressure Model

As proposed in the SESC technique [17], the whole-body centre of mass (CoM), $C_{M}={\left[\begin{array}{ccc} C_{M|x} & C_{M|y} & C_{M|z} \end{array}\right]}^{T}\in R^{3}$ of any branched chain (as our human model) can be modeled by the geometric parameters (i.e., CoM, mass and length of each link) of the original whole-body structure

(9) $$C_{M}=x_{0}+B\Phi ,$$

where $x_{0}$ is the position of $\Sigma_{0}$, matrix $B=\left[\begin{array}{ccc} A_{0} & \cdots & A_{n} \end{array}\right]\in R^{3\times 3(n+1)}$ includes the *i*-th link rotation matrices $A_{i}\in SO\left(3\right)$ with respect to $\Sigma_{W}$. Matrix $\Phi ={\left[\begin{array}{ccc} \phi_{0}^{T} & \cdots & \phi_{n}^{T} \end{array}\right]}^{T}\in R^{3(n+1)}$ contains the vector of SESC parameters $\phi_{i}\in R^{3}$, which represents the mass distribution of the human model.

To identify the unknown parameters, the whole-body CoM can be written in regressor form as

(10) $${}^{0}C_{M}=C_{M}-x_{0}=B\Phi ,$$

where ${}^{0}C_{M}$ is the CoM represented in $\Sigma_{0}$. The identification of the parameter vector Φ in such a form can be considered as a classical least-squares problem. In this work, the rotation matrices in B and the human base frame position vector $x_{0}$ are obtained from the measurements of an external motion-capture system. On the other hand, the CoM vector cannot be measured directly from the sensors but it is possible to achieve the ground-projected CoM, which corresponds to the CoP in the static condition. The whole-body CoP vector $C_{P}={\left[\begin{array}{cc} C_{P|x} & C_{P|y} \end{array}\right]}^{T}\in R^{2}$ with respect to the $\Sigma_{W}$ can thus be collected using an external force platform.

Accordingly, the least-squares problem can be solved using the stacked matrices $B^{*}$ (the superscript ${\left(.\right)}^{*}$ symbolises the pre-multiplication of the projection onto the ground plane) and vectors ${}^{0}C_{P}$ (the CoP represented in $\Sigma_{0}$) for a set of *p* human poses defined as $W\in R^{2p\times 3(n+1)}$ and $\Omega \in R^{2p\times 1}$, respectively. The vector of the identified SESC parameters $\hat{\Phi }\in R^{3(n+1)}$ can then be computed as

(11) $$\hat{\Phi }=W^{+}\Omega ,$$

where $W^{+}={\left(W^{T}W\right)}^{-1}W^{T}$ is the Moore-Penrose generalised inverse. In static conditions, we can obtain the CoP by projecting the whole-body CoM estimated by (9) with the identified SESC parameters (11) onto the ground. Then, the *x*-coordinate of such CoP can be used in (4) to obtain $x_{a_{h}|x}$.

## 3. Overloading Joint Torque

We recently proposed an algorithm to estimate the overloading torques induced into the human body joints by an external load [16]. In this work and in subsequent ones, the technique was utilised with the assumption of a known and fixed load application point. However, by employing the contact point detection method proposed in this paper, the overloading torques can be computed with much more flexibility. A brief summary of the method, with extension to the case of varying load application point, is presented in this section.

The method is based on the displacement of the CoP, computed from the difference between an estimated one, ${\hat{C}}_{P_{wo}}$ (using the procedure explained in Section 2.1), and a measured one, $C_{P_{wt}}$, employing an external sensor system. If no external interactions of the human with the environment (or with an object) occur, the estimation of the CoP vector ${\hat{C}}_{P_{wo}}$ obtained by the human body model is almost equal to the measured one $C_{P_{wt}}$. Otherwise, if any external load is applied on the human body, the estimated and the measured CoP vectors differ and the overloading torque vector varies accordingly.

By employing Equation (1), the overloading torque vector $\tau_{wo}$ without considering any external force except the ground reaction force (i.e., the body weight) can be computed by using ${\hat{C}}_{P_{wo}}$ and the vGRF vector $f_{wo}$ as

(12) $$S^{T}\tau_{wo}=\tau_{b}-\sum_{i=1}^{n_{f}}J_{{\hat{C}}_{P_{wo}i}}^{T}f_{wo,i}.$$

Similarly, the overloading torque vector $\tau_{wt}$ considering the effect of any external force, can be computed by using $C_{P_{wt}}$ and the vGRF vector $f_{wt}$ as

(13) $$S^{T}\tau_{wt}=\tau_{b}-\sum_{i=1}^{n_{f}}J_{C_{P_{wt}i}}^{T}f_{wt,i}-\sum_{j=1}^{n_{h}}J_{a_{hj}}^{T}f_{h,j},$$

where $n_{f}$ is the number of contact forces exchanged with the ground. $f_{wo}$ and $f_{wt}$ are the vGRF vectors applied at the CoP without and with the effect of external forces, as explained in Section 2.1. $n_{h}$ is the number of contact points where the external loads are applied. In this work, a single contact point $n_{h}=1$ is considered as said in Section 2.1. The contact Jacobians at the CoP vectors $J_{C_{P}}$ have fixed parameters, while the Jacobian $J_{a_{h}}$ at the contact points $x_{a_{h}}$ varies, depending on which body link the load is applied and on the application point position of the load in such a body link, so it is a function of q and $x_{a_{h}}$. Deriving from (12) and (13), the overloading joint torques can be defined as

(14) $$\Delta \tau_{s}=S^{T}\left(\tau_{wt}-\tau_{wo}\right)=-J_{{a\;}_{h}}^{T}f_{h}+\sum_{i=1}^{n_{f}}\left(J_{{\hat{C}}_{P_{wo}i}}^{T}\zeta_{i}f_{h}-J_{\Delta C_{Pi}}^{T}f_{wt,i}\right),$$

with the Jacobian of the CoP displacement defined as $J_{\Delta C_{Pi}}=J_{C_{P_{wt}i}}-J_{{\hat{C}}_{P_{wo}i}}$. It should be noted that $\tau_{b}$ does not affect the overloading torque vector $\Delta \tau_{s}$ in any body configuration since the external load effect is included in $\Delta \tau_{s}$. By disregarding $\tau_{b}$, the number of human model parameters to be identified can be considerably reduced [3]. $0\leq \zeta_{i}\leq 1$ are the distribution gains for vGRF which can be calculated from the body configuration [28,29] (N.B. $\sum_{i}\zeta_{i}=1$ is a necessary condition).

To provide the humans with an online feedback of the overloading torques during the experiment, a graphical interface was proposed in [30]. In this work, such interface was extended to additionally include the estimated position of the external contact point on the body.

## 4. Experimental Analysis

In this section, we present an experimental analysis to validate the proposed contact point detection method. First, a simulation study is performed with Simscape Multibody^TM^ (formerly SimMechanics^TM^), a multibody simulation environment for 3D mechanical systems developed and commercialised by MathWorks, Inc. (Natick, Massachusetts, USA). Then, experiments using data collected with sensor systems are carried out on two human subjects as a proof-of-concept, to test the detection capability of the technique in real environments for 3 different contact points. In addition, the estimation of the overloading torque induced on the human body joints by the external load is performed, showing a possible application of the proposed technique. The whole experimental procedure was conducted in accordance with the Declaration of Helsinki and the protocol was approved by the ethics committee Azienda Sanitaria Locale Genovese (ASL) N.3 (Protocol IIT_HRII_001).

### 4.1. Experimental Setup

**Figure 3:**
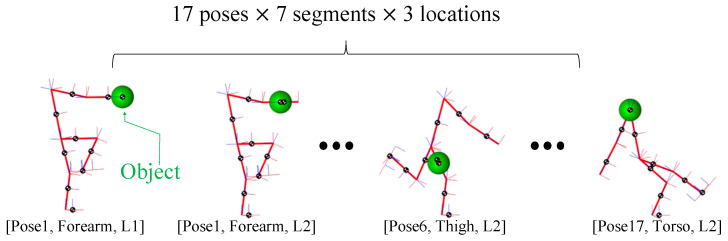
Some examples of the object placement conditions employed for contact point estimation in the simulation performed with SimMechanics™ and the proposed technique.

Simulation experiments were destined and developed to evaluate the *feasibility* of the proposed technique in localisation of the external loads on body links on different body postures. A total of 357 conditions (17 body poses × 7 segments × 3 locations) were simulated to analyse the accuracy of the method in locating the contact points. In fact, these simulations were to understand the expected performance of the proposed technique, prior to the real experiments that revealed the exact performance, as outlined in Section 4.2. The simulation was conducted, as stated above, using Simscape Multibody^TM^ and integrated using the ode15s solver. Computations were carried out using a personal computer with an Intel core i7, CPU 2.50 GHz processor, 16.0 GB 1600 MHz RAM. The state variables (e.g., joint angles) were collected from a SimMechanics^TM^ sensing model. On the other hand, $C_{p}$ and f of the human model were obtained by means of four virtual force sensors, which were located at the bottom of the foot link. To consider feasible and realistic human postures, the data to simulate such body configurations were collected through the Xsens MVN Biomech suit. For each body configuration, 3 fixed points on each one of the 7 links (proximal, central, distal) were assumed to be, one by one, the load application point. In Figure 3, a few of these cases are presented as an example.

In each of these 17×7×3 conditions, the estimated and measured CoP and vGRFs were simulated and the contact point was computed as explained in Section 2.1. It should be pointed out that in the simulation study the contact point link was assumed to be known, only the estimation error of the proposed technique is evaluated here.

**Figure 4:**
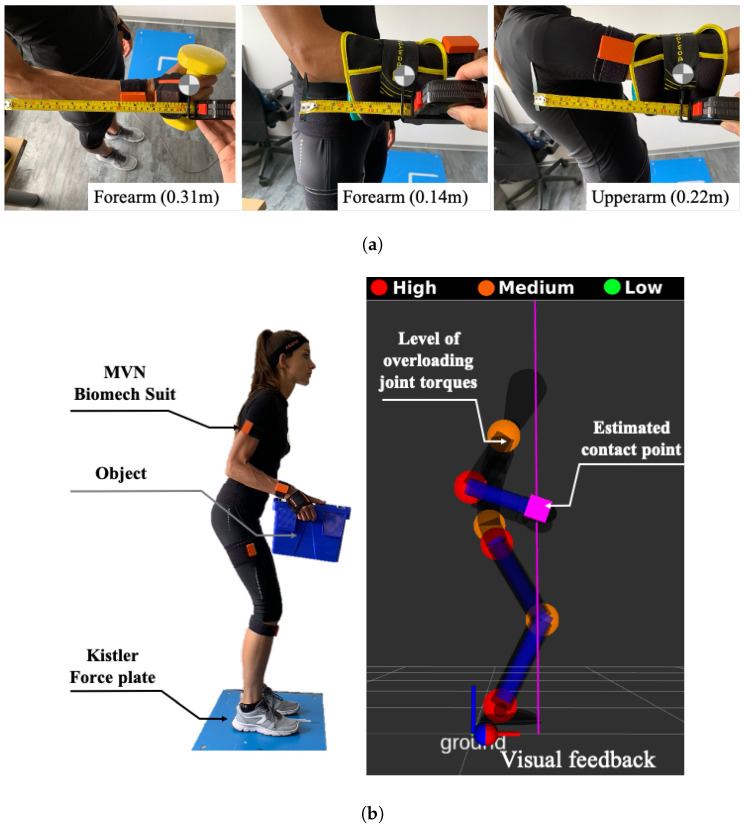
Illustration of the experimental setup. (**a**) To simulate the external load a weight was placed in 3 different body locations. (**b**) the subject was equipped with two sensor systems (a Xsens MVN Biomech suit and a force plate) and provided with a visual feedback showing the current status: the estimated contact point and the level of the joints overloadings.

For the experiments on human subjects, two healthy adults, one female and one male, labeled as subject 1 and subject 2, respectively, (age: 28 and 34 years; mass: 50 and 76 kg; height: 171 and 178 cm) were involved in the experimental session. An Xsens MVN Biomech suit, commercialised by Xsens Technologies B.V. (Enschede, Netherlands), provided with 17 inter-connected inertial measurement unit (IMU) sensors, was used to measure the whole-body motion. The whole-body CoP and vGRF were collected using a Kistler force plate, commercialised by Kistler Holding AG (Winterthur, Switzerland), as illustrated on the left side of Figure 4b. The experimental procedure included an off-line calibration experiment, a validation experiment and a demonstration experiment.

In the calibration experiment, the subjects were required to hold 25 static poses to build a suitable input data set for the CoP model parameters identification described in Section 2.2. During the acquisition, the postures were chosen by the subjects arbitrarily, but with the requirement to change the orientations of each segment as much as possible in between, to obtain variables as linearly-independent as possible. The motion was limited to the sagittal plane since a human sagittal model is considered in this work. Accordingly, both the estimated and measured CoP employed in the computations were projected onto such a plane. Once the SESC parameters were identified off-line using the data collected during the calibration experiment, they could be used to estimate the human whole-body CoP in any body configuration.

For the validation experiment, the subjects were asked to perform 3 sessions. In each session, a 4 kg weight was placed in one of the 3 pre-selected positions on the human body—hands, mid-forearms and mid-upper arms—as shown in Figure 4a, distributed equally on the two limbs (2 kg for each one). The measured linear position of the contact points in the correspondent link reference frame $\Sigma_{\mathrm{link}}$, considered to be the real ones, were respectively: 0.31 m w.r.t forearm reference frame $\Sigma_{\mathrm{FA}}$, 0.14 m w.r.t $\Sigma_{\mathrm{FA}}$ and 0.22 m w.r.t upper arm reference frame $\Sigma_{\mathrm{UA}}$. Then, the subjects were required to move freely in the sagittal plane in a quasi-static way and performing symmetrical movements with both the upper and lower limbs for the entire duration of the experiment. Meanwhile, the contact point $x_{a_{h}}$ was detected using the proposed algorithm 1. A significance level of 0.05 (5%) was selected to set both the Anderson-Darling test critical value and the $z^{*}$ value to compute the confidence interval. The corresponding values are given in Table 1 along with the confidence interval threshold, which was set arbitrarily (a further discussion about the value of $\delta_{m,threshold}$ will follow in Section 4.2).

For each experimental condition, the percentage errors between the real and the estimated linear positions of the contact point w.r.t $\Sigma_{\mathrm{link}}$ were calculated and the time needed to detect the contact point was measured.

During the demonstration experiment, the subjects were asked to move freely in the sagittal plane in a quasi-static way and performing symmetrical movements with the upper and lower limbs while holding a 5 kg box. The experimental session was divided into two phases. In the first phase, the contact point $x_{a_{h}}$ was detected. In the second phase, once the contact point position was found, the overloading torque vector on the human joints was computed using (14), by varying the contact point Jacobian $J_{{a\;}_{hj}}$ depending on the contact point position $x_{a_{h}}$ estimated. Throughout the experiment, the subjects were provided with visual feedback showing the current body configuration, the position of the estimated contact point and the overloading torque on the more meaningful joints, as illustrated in the right side of Figure 4b. Please refer to the supplementary multimedia file for the video of these experiments.

### 4.2. Results

**Figure 5:**
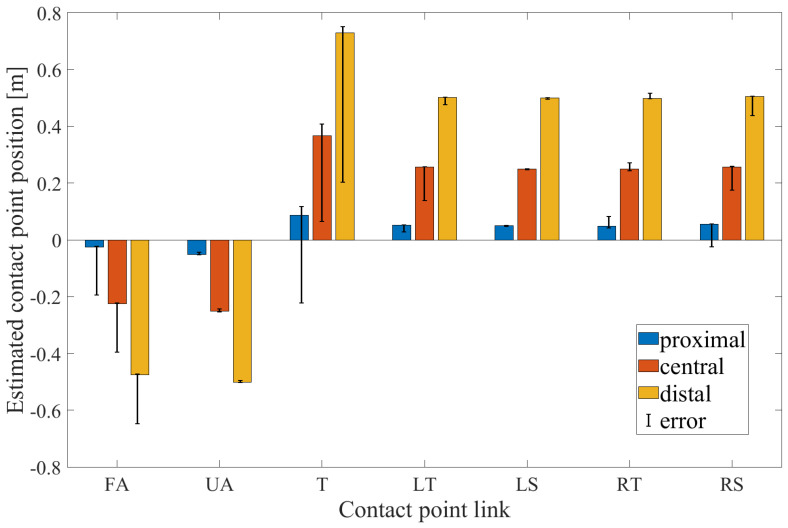
The results of the contact point estimation using SimMechanics^TM^ and the proposed technique in forearm (FA), upper arm (UA), torso (T), left thigh (LT), left shank (LS), right thigh (RT), right shank (RS) links, are illustrated in error bar plots.

The results of the simulation study to estimate the contact point position using the proposed technique are shown in Figure 5.

The colored bars represent the mean value among all the simulated body configurations of the contact point computed through the proposed technique, in the proximal (blue), central (orange) and distal (yellow) location on the link, respectively, for each link. The considered links are forearm (FA), upper arm (UA), torso (T), left thigh (LT), left shank (LS), right thigh (RT), right shank (RS). The black line superimposed on the bars represents the maximum and minimum error of the contact point position estimation between all the body configurations. In each condition, the percentage errors between the expected and the computed contact point were calculated and the mean and the standard deviation values, computed among all the contact point positions and body configurations, were obtained for each link. Specifically, the results of such computations are: 17.53% ± 20.56% for the forearm, 0.95% ± 2.71% for the upper arm, 7.18% ± 21.44% for the torso, 2.47% ± 8.98% for the left thigh, 0.62% ± 0.85% for the left shank, 2.32% ± 10.07% for the right thigh and 4.35% ± 14.68% for the right shank, respectively.

Since static body postures were considered in the simulation study, a simple computation of the contact point using (4) and the knowledge about the contact link were sufficient to accurately identify the contact point position $x_{a_{h}}^{N}$. Nevertheless, as explained in Section 2.1, a criterion to identify the contact link must be set without a priori knowledge thus the Anderson-Darling normality test is employed. Moreover, to ensure a good level of accuracy with real data, which are inevitably affected by artifacts and noise, the concept of confidence level $\delta_{m}$ is introduced.

The results of the experiments on human subjects are hereafter presented. As regards the validation experiment, the percentage error of the contact point positions and the time needed to estimate it, in each experimental condition, are shown in Table 2, for both subject 1 and 2.

**Figure 6:**
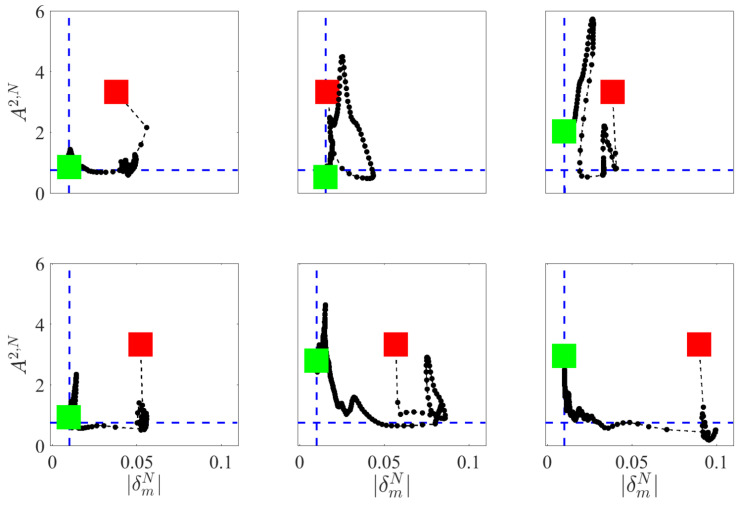
Results of the validation experiment: $A^{2}$ and $\delta_{m}$, on the *y*-axis and on the *x*-axis respectively, computed for subject 1 (first row) and subject 2 (second row), in each experimental condition, for the correspondent contact point link: hands (left column), mid-forearms (middle column) and mid-upper arms (right column).

The changes of $A^{2,N}$ and $\delta_{m}^{N}$ during the contact point detection phase, on the *y*-axis and on the *x*-axis respectively, are illustrated in Figure 6.

The red square represents the starting point while the green square represents the instant in which the contact point is detected. The contact point link is selected as soon as $A^{2,N}<A_{critical}^{2}$ meaning that the hypothesis of normality for the data set of the contact point link can be accepted. Nevertheless, the contact point link is not changed even if the value of $A^{2,N}$ return beyond the threshold. In fact, it should be noted that in some cases, in the end point, $A^{2,N}>A_{critical}^{2}$. The contact point position is then found when the value of $\left|\delta_{m}^{N}\right|$ is under the threshold $\left|\delta_{m,threshold}\right|$. This means that the confidence level of the mean value is included within the predefined threshold. In all the experimental conditions, even if the condition $n^{j}>50$ was satisfied and values $A^{2,j}$ were computed also for other candidate links, the link that satisfies first the condition $A^{2,N}>A_{critical}^{2}$ was always the actual contact point link.

**Figure 7:**
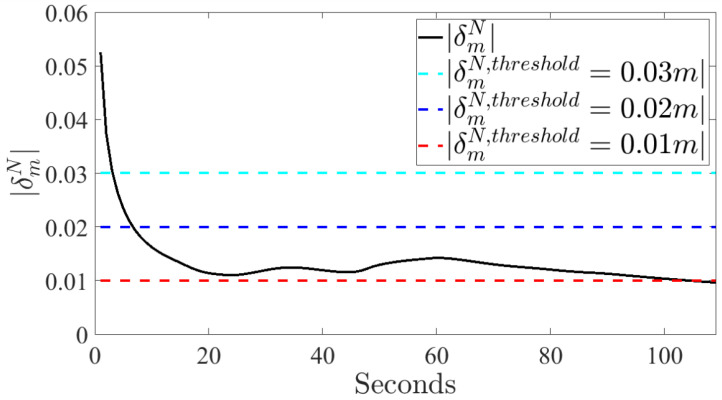
Illustrative trend of $\delta_{m}^{N}$ during the validation experiment represented with varying values of $\delta_{m,threshold}$.

Finally, typical results of the correlation between the choice of $\delta_{m,threshold}^{N}$ and estimation time are shown in Figure 7.

By setting a higher threshold, the contact point position could be found faster, nevertheless, with a larger confidence interval thus with less accuracy. Hence, $\left|\delta_{m,threshold}^{N}\right|$ has been set equal to 0.01 m as a good compromise between time and accuracy.

**Figure 8:**
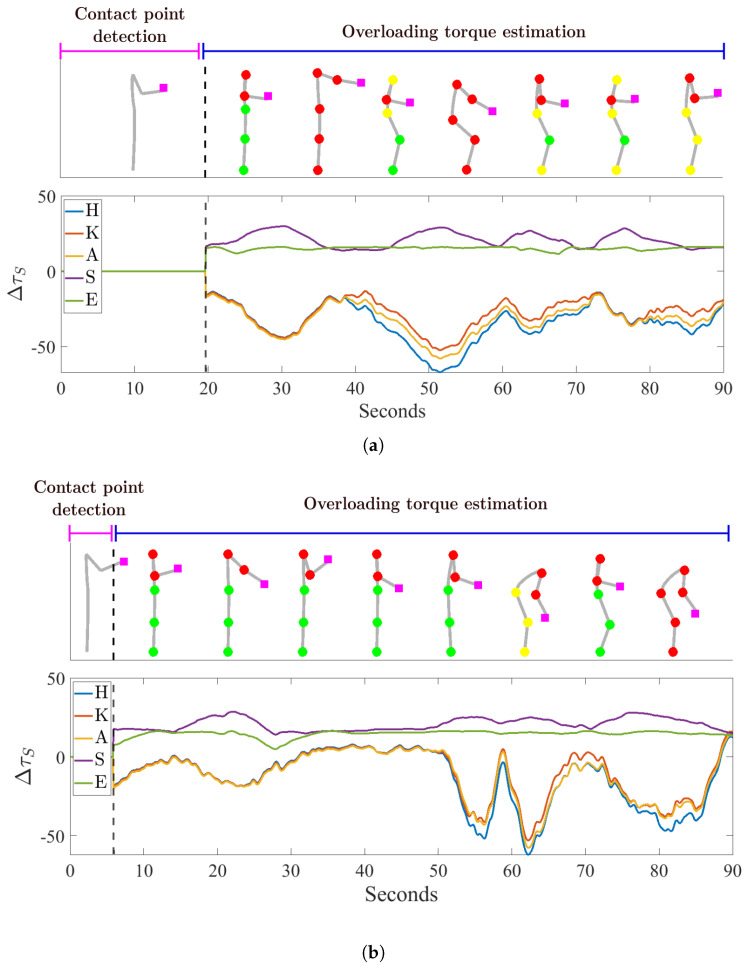
Results of the demonstration experiment to show the application of the proposed contact point detection method in the estimation of joint overloading. For subject 1 (**a**) and for subjects 2 (**b**), respectively, the human kinematic and dynamic status in some illustrative instants throughout the experiment (upper graph) and the overloading torques induced by the external load (lower graph) are represented.

In Figure 8 the results of the demonstration experiments are illustrated for subject 1 (Figure 8a) and for subject 2 (Figure 8b), respectively.

As explained in Section 4.1, the experiment was divided into two phases: the contact point detection and the overloading joint estimation. A black dotted line separates the two phases in the graphs. For each subject, two graphs are reported. In the upper graph, the 2-D stick-model of the human is depicted in some illustrative body configurations throughout the experiment. Only during the second phase, the joint torques, which are computed based on the contact point estimated in the first phase, are displayed in the form of spheres superimposed on the stick-model, color-coded to denote a high (red), medium (yellow) or (low) level of joint overloading. The 3 levels of overloading are determined as explained in Table 3.

The contact point position is depicted as well through a pink square. This information was provided online to the subject by means of the graphical interface during the experiment. The percentage error of the contact point positions and the estimation time are, respectively: 5.04% and 19.60 s for subject 1 and 9.83% and 5.80 s for subject 2. In the lower graph, the overloading torque values for the human body model joints: hip (H), knee (K), ankle (A), torso (T), shoulder (S) and elbow (E) are shown. In the first phase, before the black dotted line, the values are zero since the contact point is not found yet while in the second phase, after the black dotted line, it is possible to compute them. As explained in Section 4.1, only symmetrical movements were performed in this experiment thus the information of the two legs and arms are considered to be equal.

## 5. Discussion

In this Section we first discuss the results of the simulation study and then we address the results of the experiments performed on human subjects. For the simulation study, results indicated that the estimation error of the contact point position, computed between all the body configurations considered, was significantly low in most of the links. However, some exceptions were found for the torso and the forearm. This is most likely due to a failure in computing the intersection between the perpendicular line passing through $x_{a_{h}|x}$ and the link segment. In fact, when the vertical projection lies slightly beyond the link end-point, the contact point position can not just be detected and this lead to a misleading increment of the percentage error. Accordingly, we can state that the proposed method is able to accurately estimate the contact point position in most of the cases, with a low chance of failure occurring in extreme conditions (i.e., in the proximity of the link end-points).

An analysis of the performance of the proposed method in real settings indicated that, some factors such as movement artifacts (that are present even during quasi-static motion) and the noise introduced by sensor systems, could affect the accuracy of the estimation. To compensate for this effect in this work, a statistical analysis approach was introduced with the aim to improve the accuracy of the estimation. The link was correctly identified in all three experimental conditions, in the validation experiment, as well as in the demonstration experiment, for both subjects. It should be noted that the load was not applied punctually on the human body in any of the experimental conditions. However, in this work, it is assumed that the contact point was the mid point of the load application area. Considering that for the purposes of our framework the contact point detection is needed to set the overloading joint torque estimation, such an assumption is deemed acceptable. Furthermore, the localisation accuracy is, in our opinion, sufficiently good for the industrial applications we target. Considering the time needed to detect the contact point, in both the validation and the demonstration experiment, less than half a minute was required, proving the ’industrial-fast’ detection capacity of the proposed system. Accordingly, we can state that the proposed method shows a promising potential to quickly detect the contact point position, thus, we can assume that the overloading torque computed on the basis of the estimated contact points are good indicators for the human effort while performing activities with an external load. Consequently, their minimisation by means of an optimisation procedure can contribute to guide the humans toward more ergonomic body configurations thus addressing healthier working condition. It should be pointed out that, due to the assumption of quasi-static, sagittal and symmetric movements and the capability of the method to work only with vertical forces, the proposed framework can be deployed in a certain class of industrial tasks, which are quite numerous (lifting, carrying, pick and place in sagittal plane, etc.).

## 6. Conclusions

In this paper, we proposed a novel method to approximately detect the contact point when an external force is exerted on the human body, based on the estimation of the CoP and on the measurements collected by a motion-capture system and a force plate. Given its capability to provide a rather accurate and quick online estimation of the contact point on the human body, such a method can be used prior to the monitoring of the body dynamic states such as the overloading effects. By minimising such an effect, an optimised body configuration can be found and the risk of injuries for the human body joints can be significantly mitigated. Future works will focus on the extension of the method to a more articulated human model by considering not only the sagittal plane and more complex movements. Accordingly, since the force place severely reduces the work space, it will be replaced with insole sensors that are easily wearable (by placing them inside the shoes) and do not impose any constraints on the subject’s mobility. As a result, it will be possible to apply the proposed method in real industrial settings.

## Figures and Tables

**Table 1:** Anderson-Darling test critical value, $z^{*}$ value and confidence interval threshold according to a significance level of 0.05 (5%)

$A_{\mathrm{critical}}^{2}$	$z^{*}$	$\delta_{m,\mathrm{threshold}}$ [m]
0.735	1.96	0.01

**Table 2:** Results of the validation experiment.

Subject	Experimental Condition	Percentage Error	Estimation Time
	Hands	1.022%	9.4 s
1	Mid-forearms	20.92%	16.1 s
	Mid-upper arms	11.15%	14.4 s
	Hands	1.17%	16.0 s
2	Mid-forearms	48.04%	27.2 s
	Mid-upper arms	16.05%	17.5 s

**Table 3:** Stepwise scheme for joint torque overloading level

Overloading Level	Control Threshold
GREEN	$0<\Delta \tau_{i}\leq 0.3\tau_{\max_{i}}$
ORANGE	$0.3\tau_{\max_{i}}<\Delta \tau_{i}\leq 0.6\tau_{\max_{i}}$
RED	$0.6\tau_{\max_{i}}<\Delta \tau_{i}\leq \tau_{\max_{i}}$

## Supplementary Materials

The following are available online at https://www.mdpi.com/1424-8220/20/16/4471/s1, Video S1: An Online Method to Detect and Locate an External Load on the Human Body with Applications in Ergonomics Assessment.

## Abbreviations

The following abbreviations are used in this manuscript:

CoP	centre of pressure
CoM	centre of mass
DoFs	degrees of freedom
EAWS	ergonomic assessment work-sheet
EMG	electromyography
ERC	european reasearch council
IMU	inter-connected inertial238measurement unit
SESC	statically equivalent serial chain
vGRF	vertical ground reaction force
